# Perfusion Computed Tomography as a Screening Tool for Pending Delayed Cerebral Ischemia in Comatose Patients After Aneurysmal Subarachnoid Hemorrhage: A Retrospective Cohort Study

**DOI:** 10.1007/s12028-023-01855-6

**Published:** 2023-10-11

**Authors:** Thor Löwe Busse, Sune Munthe, Baskaran Ketharanathan, Karsten Bülow, Bjarni Jóhannsson, Anabel Diaz, Troels Halfeld Nielsen

**Affiliations:** 1 Department of Neurosurgery, Odense University Hospital, Odense, Denmark; 2 Department of Anaesthesiology and Intensive Care, Odense University Hospital, Odense, Denmark; 3 Department of Radiology, Odense University Hospital, Odense, Denmark

**Keywords:** Cerebral vasospasm, Delayed cerebral ischemia, Perfusion computed tomography, Intracranial aneurysm, Subarachnoid hemorrhage, Hypoperfusion, Screening, Brain hypoxia, Neurointensive care, Comatose

## Abstract

**Background:**

Aneurysmal subarachnoid hemorrhage (aSAH) is frequently complicated by delayed cerebral ischemia (DCI), leading to poor outcomes. Early diagnosis of DCI is crucial for improving survival and outcomes but remains challenging in comatose patients. In this study, we aimed to evaluate computed tomography with angiography and perfusion (P-CT) as a screening modality on postictal days four and eight for impending DCI after aSAH in comatose patients using vasospasm with hypoperfusion (hVS) as a surrogate and DCI-related infarction as an outcome measure. Two objectives were set: (1) to evaluate the screening’s ability to accurately risk stratify patients and (2) to assess the validity of P-CT screening.

**Methods:**

We conducted a retrospective review of the records of comatose patients with aSAH from January 2019 to December 2021 who were monitored with P-CT scans on days four and eight. The event rates of DCI-related infarction, hVS, and endovascular rescue therapy (ERT) were analyzed, and the sensitivity, specificity, negative predictive value (NPV), and positive predictive value (PPV) for DCI were calculated. DCI-related infarction was defined as new secondary cerebral infarction > 48 h < 6 weeks post aSAH not attributable to other causes, and hVS was defined as arterial narrowing with corresponding hypoperfusion on P-CT.

**Results:**

Fifty-six comatose patients were included, and 98 P-CT scans were performed. The incidence of DCI-related infarction was 40%. Screening P-CT on days four and eight found vasospasm in 23% of all patients, including 11% with hVS. A positive hVS on day four or eight revealed a relative risk of 2.4 [95% confidence interval (CI) 1.13–5.11, *p* = 0.03], sensitivity of 23% (95% CI 8–45, *p* = 0.03), specificity of 95% (95% CI 36–100, *p* = 0.03), PPV of 0.83 (95% CI 0.36–1.00, *p* = 0.03), and NPV of 0.65 (95% CI 0.50–0.78). Six positive P-CT scans led to digital subtraction angiography in five patients, three of whom received ERT. All ERT-intervened patients developed DCI-related infarction.

**Conclusions:**

P-CT resulted in few interventions and often resulted in late detection of DCI at an irreversible stage. Although a positive P-CT result accurately predicts impending DCI-related infarction, screening on days four and eight alone in comatose patients with aSAH often fails to timely detect impending DCI. Based on our analysis, we cannot recommend P-CT as a screening modality. P-CT is likely best used as a confirmatory test prior to invasive interventions when guided by continuous multimodal monitoring; however, prospective studies with comparison groups are warranted. The need for a reliable continuous screening modality is evident because of the high rate of deterioration and narrow treatment window.

## Introduction

Delayed cerebral ischemia (DCI) is the leading preventable cause of poor outcome in patients with aneurysmal subarachnoid hemorrhage (aSAH) [1–5]. Prompt diagnosis of incipient DCI is vital to improve outcome and survival in patients with aSAH, as progression to irreversible DCI-related infarction can be averted with timely intervention. However, early diagnosis remains a challenge [2].

DCI is commonly linked to vasospasm [6–8], as vasospasm frequently occurs and can reduce blood flow, resulting in hypoperfusion and DCI. However, DCI can occur without vasospasm, and vasospasm can occur without DCI, highlighting a complex interplay rather than a simple cause-and-effect relationship between these two conditions [6, 8–13]. Therefore, only symptomatic vasospasm should be treated as a surrogate for impending DCI [14].

In comatose patients, the detection of impending DCI is further complicated by greatly impeded options for neurological examination. Repeated neurological examination is important for screening and subsequently distinguishing between symptomatic and nonsymptomatic vasospasm [15, 16]. However, in comatose patients, this requires briefly lowering the sedation, which may not always be feasible in poor-grade patients [17]. Additionally, the choice of sedative plays a significant role in these situations. For instance, midazolam, because of its longer half-life, can result in a protracted wake-up time compared to propofol, further complicating the timing and effectiveness of the neurological examination [18].

Debate continues about the best strategies for monitoring comatose patients with aSAH. DCI-related infarction can eventually be diagnosed on scans, but these scans fail to diagnose the ongoing injurious process for which interventions are available [16]. Therefore, vasospasm with hypoperfusion (hVS) detected on computed tomography angiography and perfusion (P-CT) can be used as a surrogate marker for impending DCI in lieu of symptomatic vasospasm [19–22]. Recent studies suggest that P-CT may be incorporated as a screening tool for DCI, especially on days four to eight post ictus with the highest a posteriori risk [23–25].

However, literature on P-CT screening in comatose patients is scarce and has failed to show a decreased incidence of DCI and improved patient outcome despite increased detection and intervention. Furthermore, the ability of the modality to timely diagnose and risk stratify patients remains unknown [20, 26–29]. If P-CT screening detects impending DCI, more timely targeted interventions can be initiated. On a similar note, if a negative screening is a reliable prognostic indicator for not developing DCI-related infarction, it would allow better stratification of patients and focus prophylactic measures and rescue therapies on patients who would likely see the most significant benefit from them [2].

In this retrospective cohort study, we aimed to evaluate the effectiveness of P-CT as a screening tool on days four and eight for detecting impending DCI in comatose patients after aSAH. The study had two specific objectives:

1. To evaluate the screening’s ability to risk stratify by calculating the negative predictive value (NPV) of patients with inconspicuous screening results who did not develop DCI-related infarction and comparing the negative post-test probability of developing DCI-related infarction to the background incidence in the existing literature. Sensitivity, specificity, and positive predictive value (PPV) were also estimated to comprehensively evaluate the screening’s performance.
2. To assess the validity of P-CT screening by determining the proportion of patients receiving interventions for vasospasm and hVS identified on the screen and analyzing their outcomes. This would help understand the clinical implications of the identified vasospasm and hypoperfusion.

The primary outcome measures were the proportion of positive screens, interventions resulting from screening, and DCI-related infarction occurring within 6 weeks post ictus.

## Methods

### Study Population and Study Design

We conducted a retrospective cohort study evaluating P-CT on days four and eight as a screening modality for impending DCI in comatose patients with confirmed aSAH admitted to the neurointensive care unit at Odense University Hospital, Denmark, between January 2019 and January 2022. Coma was defined as prolonged unconsciousness in which the patient is unresponsive to external stimuli and cannot awaken spontaneously. Specifically, patients with a Glasgow Coma Scale (GCS) score of 3 on days four and/or eight due to sedation were considered comatose, regardless of initial GCS score. As part of the standard protocol, these patients underwent P-CT (standard cerebral computed tomography [CTC], perfusion, and carotid angiography [CTA] scans) on days four and/or eight after ictus. Demographic, clinical, and radiographic patient characteristic data were retrospectively collected. Patients aged < 18 years and pregnant patients were excluded from the study. The ethics committee of Southern Denmark was consulted for this study and concluded that no ethics approval was needed (ID 20222000-3).

### Patient management

Patients were treated according to the Danish national guidelines for subarachnoid hemorrhage (SAH) management [30] with endovascular or microsurgical aneurysm treatment < 48 h after ictus, extraventricular drain in cases of elevated intracranial pressure (ICP), and intubation and mechanical ventilation when the GCS score was < 8. Coma was achieved using propofol or midazolam, with the addition of thiopental when needed. Propofol was initially used for ventilator management and was substituted for midazolam if prolonged sedation was required. Thiopental was introduced as an additional measure for ICP control in patients with ICP exceeding 20 mm Hg that could not be mitigated by other interventions, such as deep sedation, hypertonic saline, and/or head elevation. Patients were kept normovolemic and were given 60 mg of oral nimodipine every 4 h if tolerated by vital parameters. Cerebral blood flow velocity screening was performed daily with transcranial Doppler (TCD) in comatose patients, followed by angiographic confirmation of vasospasm when the middle cerebral artery mean flow velocity exceeded 120 cm/second. At our institution, P-CT scans were performed on days four and eight in comatose patients. The day eight scan was omitted if arousal between day four and day eight was sufficient for neurological assessment. Additional P-CT scans and other types of scans were performed on clinical indications. In patients with P-CT-confirmed vasospasm, the therapeutic aim was to maintain a systolic blood pressure exceeding 160 mm Hg. Nimodipine treatment was upheld at its maximum dosage unless it was required to be curtailed because of high doses of norepinephrine. If hypoperfusion and vasospasm were present despite induced hypertension, digital subtraction angiography (DSA) was performed, followed by endovascular rescue therapy (ERT) with intraarterial nimodipine and/or balloon angioplasty.

### Screening Protocol

All patients with aSAH were screened for hVS with a P-CT scan on days four and eight if comatose. A P-CT scan consists of a standard CTC scan, a CTA scan, and a perfusion scan. All P-CT scans were acquired on a GE Revolution 256-slice scanner.

CTC scans were firstly performed (parameters: 120 kV, noise index [NI] = 6.2, 30–900 mA, 0.625–5-mm slice reconstruction), followed by 60 ml of contrast (Omnipaque 350 mg iodine/ml at an infusion speed of 5 ml/second) and a bolus of 30-ml saline solution for the CTA scan (parameters: 100 kV, NI = 8, 30–1,080 mA, 0.625–2.5-mm slice reconstruction). For the perfusion scan, an additional 40 ml of contrast (Omnipaque 350 mg iodine/ml at an infusion speed of 4 ml/second) followed by a 30-ml saline bolus was injected, and the scan was performed (parameters: 80 kV, NI = 8, 20–300 mA, 5-mm slice reconstruction). All scans were interpreted by neuroradiologists.

### Variables and Definitions

Descriptive variables, including demographic, clinical, and radiological data, were collected from patient charts. The world federation of neurological surgeons (WFNS) grade and Hunt and Hess score represented the initial assessment at admission (day 0). All included patients were comatose (GCS score = 3) on days four and/or eight because of sedation.

Outcome variables, including hVS parameters, ERT, and DCI-related infarction, were collected from patient charts and scans and used for statistical and event-rate analyses. The original interpretation and description of the scans were conducted by neuroradiologists. During the data-collecting process for this retrospective study, scans were revisited by a vascular neurosurgeon specifically to identify and exclude any infarctions that could be attributed to causes other than DCI. Infarction attributable to causes other than DCI were omitted.

P-CT examinations were defined as “screen” if conducted on days four and/or eight as mandated by our internal protocol for managing unconscious patients with aSAH. P-CT was defined as “additional” if conducted on non-predefined dates, i.e., scans initiated because of atypical clinical findings indicating impending DCI. These findings could include abnormal TCD results, unexpected ICP elevations, or difficulties in weaning the patient off sedation. Screen P-CT scans and additional P-CT scans were analyzed separately.

hVS-positive P-CT scans were defined as vasospasm with concomitant hypoperfusion. Vasospasm was defined as narrowing of one or more vessels by > 50% compared to adjacent segments, extradural segments, corresponding contralateral arteries, or prior scans. Hypoperfusion was defined as a cerebral blood flow reduction of > 50% compared to a prior scan, or to the contralateral corresponding vascular territory or the ipsilateral adjacent vascular territory on the perfusion scan, that was not attributable to other causes [15, 24, 31].

DCI-related infarction was defined as at least one new secondary cerebral infarct presenting < 6 weeks after aneurysm rupture not present on a computed tomography (CT) scan performed < 48 h post aneurysm-securing procedure and not attributable to other causes [16].

### Statistics

Continuous patient characteristics were expressed as median with interquartile range or mean ± SD, as appropriate. Discrete patient characteristics were expressed as percentages. Screen P-CT was included as a binary variable (positive/negative) for hVS, hypoperfusion, and vasospasm. DCI-related infarction was likewise included as a binary variable.

For specific objective 1, a contingency table was used to calculate relative risk, using P-CT results as the intervention variable and DCI-related infarction as the outcome variable. Using a two-sided Fisher’s exact test, the intervention (positive screen) group was compared to the nonintervention (negative screen) group. The risk-stratifying capabilities of P-CT screening on days four and eight were evaluated using sensitivity, specificity, PPV, and NPV. The NPV was particularly important, as interventions for vasospasm or hVS did not bias the outcome measure. The negative post-test probability was extrapolated from the NPV and compared with the incidence of DCI. A stratified analysis was then performed using different criteria for a positive P-CT result (hVS only, vasospasm only, or vasospasm regardless of hypoperfusion) and substratified by individual days of P-CT. This enabled us to explore the risk-stratifying capacities of P-CT screening under various hypothetical conditions.

For specific objective 2, an event-rate analysis was performed. This analytical approach measured the implications of implementing a P-CT screening protocol. Specifically, the frequency of positive P-CT scans and the resulting interventions for two conditions, vasospasm (intervened with induced hypertension) and hVS (intervened with hypertension and ERT), were analyzed alongside the outcome of DCI-related infarction. Data from this positive screen group were then juxtaposed to the rate of “silent” DCI-related infarction of the negative screen group.

This comprehensive approach aimed to provide a clearer understanding of the diagnostic accuracy and clinical implications of P-CT scans for patients at risk of DCI. Statistical analyses were performed using STATA V.17 (STATA LLC, College Station, TX). Statistical significance was set at *p* < 0.05.

### Missing Data

All patients who underwent at least one P-CT examination were included to enhance relevance to clinical practice, whereas the remaining scans were treated as missing data for patients with only one P-CT. Missing data included instances in which scans were performed only on day four or eight because of clinical deterioration after day four, clinical improvement after day four, or death. Data that could not be retrospectively recreated were also deemed missing. All missing data have been reported in the Results section.

## Results

### Study Population Characteristics

**Fig. 1:**
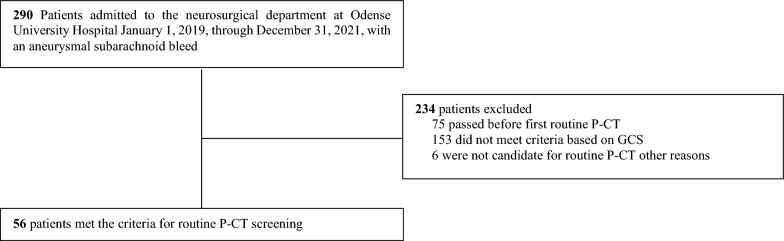
Flow diagram of retrospective cohort consisting of comatose patients with aneurysmal subarachnoid hemorrhage eligible for screening with cerebral CT scans with perfusion and angiography (P-CT) on days four and/or eight. Criteria of routine P-CT screening was comatose on day four and/or eight

Between January 2019 and January 2022, 290 patients were admitted to our department with aSAH. Fifty-six (19%) patients met the criteria for P-CT screening, being comatose for at least four days, and 98 screen P-CT scans were performed (Fig. 1).

Nine patients received one additional P-CT scan, one patient received two additional P-CT scans, and one patient received three additional P-CT scans. One patient had missing data regarding DCI-related infarction that could not be retrospectively analyzed.

**Fig. 2:**
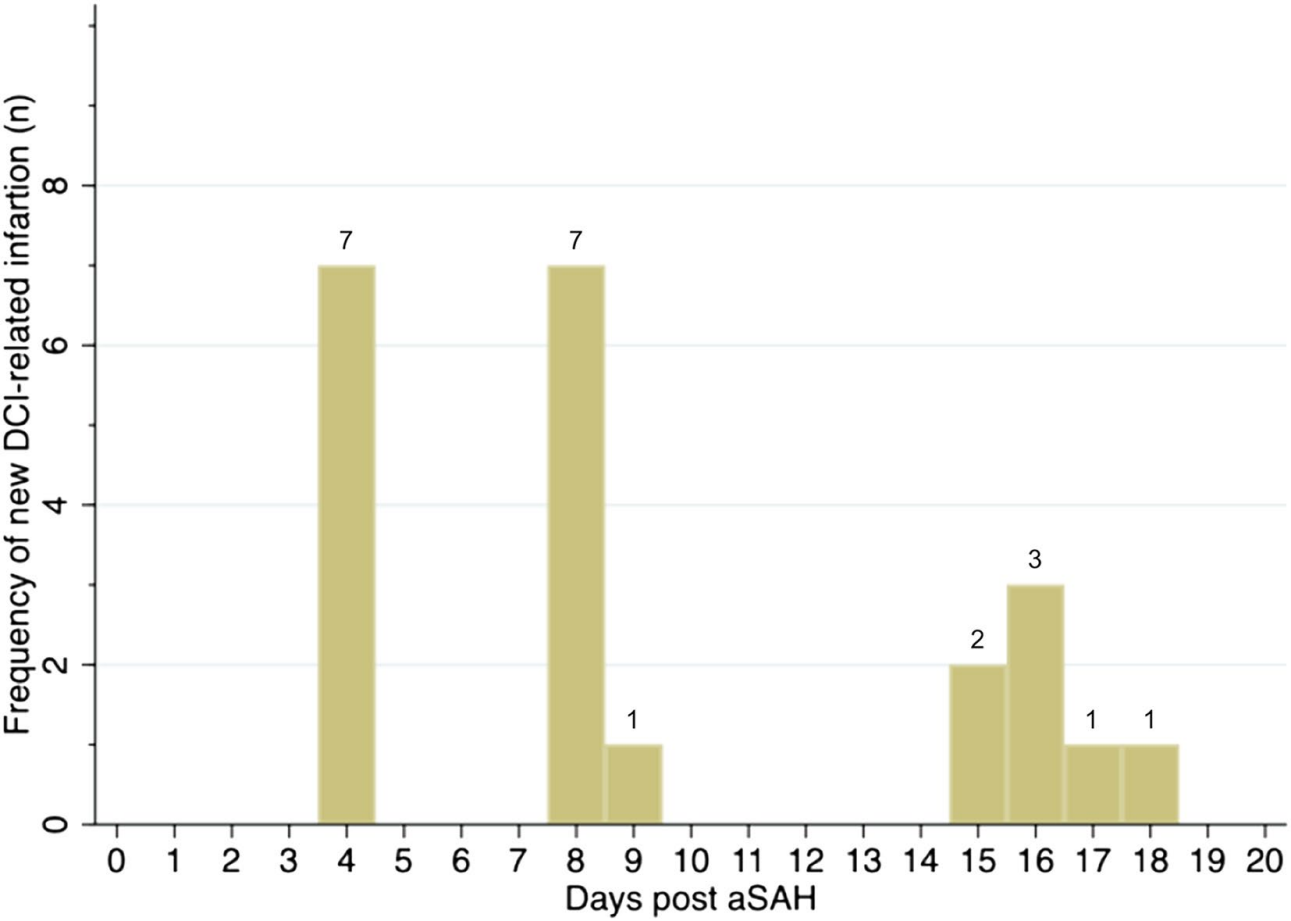
The occurrence diagnoses of delayed cerebral ischemia (DCI) related infarction (*x*-axis, number of patients) over time (*y*-axis, post-ictal days) following aneurysmal subarachnoid hemorrhage (aSAH) in 56 comatose patients based on 98 perfusion CT screenings performed on days four and/or eight, as well as additional scans on clinical indications

Detailed demographic and clinical characteristics are presented in Table 1. The majority of patients were women, and 79% of the cohort had a WFNS score of 4–5 at admission. The incidence of DCI-related infarction was 40%. Figure 2 summarizes which days DCI-related infarction was discovered. A multivariate logistic regression analysis revealed no significant associations between patient characteristics and DCI-related infarction, hVS, or vasospasm. However, DCI-related infarction was found to be significantly associated with in-hospital mortality. Eight of the ten patients who died had developed DCI-related infarction, resulting in a relative risk ratio of 6 (95% confidence interval [CI] 1.73–20.77, *p* = 0.01).

**Table 1:** Characteristics of comatose patients with aneurysmal subarachnoid hemorrhage admitted to Odense University Hospital January 2019 to December 2021, eligible for P-CT screening on days 4 and/or 8

Patient characteristic	Total, *N* = 56
Age, median (IQR)	61 (55–69)
Sex (female), *n* (%)	45 (80)
WFNS grade at admission, *n* (%)	
Grade 1–3	12 (21)
Grade 4–5	44 (79)
Modified fisher grade, *n* (%)	
Grade 1–2	5 (9)
Grade 3–4	51 (91)
Hunt–Hess grade at admission, *n* (%)	
Grade 1–3	20 (36)
Grade 4–5	36 (64)
Aneurysm location, *n* (%)	
AcomA	21 (38)
MCA	14 (25)
ICA	6 (11)
PcomA	6 (11)
Pericallosal	3 (5)
Basilar	3 (5)
VA	2 (4)
PICA	1 (2)
Aneurysm securing procedure, *n* (%)	
Endovascular	43 (77)
Days comatose, mean (SD)	13 (7.3)
In-hospital mortality	10 (18)
Day 4 P-CT, *n* (%)	
hVS detected	5 (9)
DCI-related infarctions	7 (13)
Day 8 P-CT, *n* (%)	
hVS detected	4 (9)
DCI-related infarctions	12 (28)
Intervention, *n* (%)	
ERT as result of scan	3 (5)
DCI-related infarctions on any scan, *n* (%)	
DCI-related infarctions	22 (40)
DCI-related infarctions on screen, *n* (%)	12 (55)

*AcomA* anterior communicating artery, *DCI* delayed cerebral ischemia, *ERT* endovascular rescue therapies, *hVS* vasospasm with hypoperfusion, *ICA* internal carotid artery, *IQR* interquartile range, *MCA* middle cerebral artery, *PcomA* posterior communicating artery, *P-CT* cerebral computed tomography with angiography and perfusion, *PICA* posterior inferior cerebellar artery, *VA* vertebral artery, *WFNS* World Federation of Neurological Surgeons

### Results of P-CT Screening

Ninety-eight scans were performed on 56 patients. Fourteen patients had missing data from either the day four or day eight scan.

### Detection of Vasospasm and Hypoperfusion

Vasospasm was found in a total of 13 of 56 comatose patients (23%). hVS was noted on day four (*n* = 2), day eight (*n* = 1), and both days four and eight (*n* = 3) in six patients. The other seven patients did not have hypoperfusion; their vascular narrowing was noted on day four (*n* = 1), day eight (*n* = 4), and both days four and eight (*n* = 2). One patient exhibited hypoperfusion yet did not have vascular narrowing and subsequently did not develop DCI-related infarction.

### Interventions and Outcomes of hVS-Positive Scans

A positive hVS result was followed by DSA in five patients. The decision to forego DSA in one hVS-positive patient was due to the presence of severe DCI-related infarction in other areas, which made any further intervention considered futile. Of the five patients who underwent DSA, two were not subjected to any further interventions because the DSA revealed slow and reduced blood flow without any significant vascular narrowing. DCI-related infarction was later identified in one of these two patients.

ERTs were performed on three patients as a direct result of the screening. All the intervened patients developed DCI-related infarction in the vascular territory of the intervened vessel, except patient 3’s left anterior cerebral artery. However, patient 3 had multiple hVS foci and still developed DCI-related infarction corresponding to other intervened vessels (Table 2).

**Table 2:** P-CT screening on days 4 and/or 8 of 56 comatose aSAH patients enabled ERT in three additional cases

Patient	Aneurysm location	ERT day	Spastic artery on DSA	Intervened artery	Lateralization of ERT	Intervention	Day of DCI-related infarction	Lateralization of DCI-related infarction	Vascular territory of DCI-related infarction
1	PcomA	4	MCA + ACA	ICA	Left	I.A. nimodipine 3 mg	9	Right	MCA
		8	MCA + ACA	ICA	Right	I.A. nimodipine 3 mg	9	Left	ACA
2	AcomA	8	ACA	ACA	Bilateral	I.A. nimodipine 2 mg	4	Left	PCA
							12	Bilateral	ACA
							12	Left	MCA
3	Pericallosal	4	MCA + ACA	MCA + ACA	Bilateral	Balloon angioplasty	4	Left	MCA
							4	Right	ACA
							8	Right	MCA
							8	Left	PCA

Course of progression from hVS to DCI-related infarction after ERT in comatose patients with aSAH with hVS on days 4 and/or 8 of P-CT screen. All the intervened patients developed DCI in the vascular territory of the intervened vessel, except patient 3’s left ACA. However, patient 3 had multiple hVS foci and still developed infarction corresponding to other intervened vessels

*ACA* anterior cerebral artery, *AcomA* anterior communicating artery, *aSAH* aneurysmal subarachnoid hemorrhage, *DCI* delayed cerebral ischemia,* DSA* digital subtraction angiography, *ERT* endovascular rescue therapy, *hVS* vasospasm with hypoperfusion, *I.A.* intra-arterial, *ICA* internal carotid artery, *MCA* middle cerebral artery, *PCA* posterior cerebral artery, *PcomA* posterior communicating artery, *P-CT* cerebral computed tomography with angiography and perfusion

### Performance Characteristics of P-CT

Table 3 summarizes the performance of P-CT as a screening modality for DCI-related infarction < 6 weeks in comatose patients with aSAH on days four and/or eight, presented based on different definitions of a positive test result and stratified by individual days.

**Table 3:** Performance characteristics of P-CT with angiography and perfusion screening in predicting DCI-related infarctions in a cohort of 56 comatose patients with aneurysmal subarachnoid hemorrhage

Variable (*n* patients)	Relative risk (95% CI)	Sensitivity (95% CI)	Specificity (95% CI)	PPV (95% CI)	NPV (95% CI)	*P*
Combined day 4 or 8 (*N* = 56)^a^						
Any VS (*n* = 14)	2.2 (1–4)	41% (21–64)	88% (72–97)	69% (39–91)	69% (53–82)	0.02
hVS (*n* = 6)	2.3 (1–5)	23% (8–45)	95% (84–100)	83% (36–100)	65% (50–78)	0.03
Non-hypo VS (*n* = 8)	1.7 (0.7–4)	22% (6–48)	90% (74–98)	57% (18–90)	67% (51–80)	0.21
Day 4 P-CT (*n* = 55)^a^						
Any VS (*n* = 9)	1.9 (0.9–4)	27% (11–50)	91% (75–98)	67% (30–93)	64% (49–78)	0.09
hVS (*n* = 5)	2.2 (0.9–5)	18% (5–40)	97% (84–100)	80% (28–100)	63% (48–77)	0.08
Non-hypo VS (*n* = 4)	1.4 (0.4–4)	11% (1–35)	93% (78–99)	50% (7–93)	64% (48–78)	0.48
Day 8 P-CT (*n* = 43)^a^						
Any VS (*n* = 10)	1.8 (0.9–4)	33% (13–59)	88% (68–97)	67% (30–93)	64% (45–80)	0.11
hVS (*n* = 4)	1.9 (0.8–5)	17% (4–41)	96% (79–100)	75% (19–99)	61% (43–76)	0.20
Non-hypo VS (*n* = 6)	1.7 (0.6–4)	20% (4–48)	91% (72–99)	60% (15–95)	64% (45–80)	0.30

The table is divided into three sections based on the individual days tested. The subgroups are further divided based on possible scan results, including any VS, hVS, and non-hypo VS. The table includes the number of patients (*n*) in each subgroup, as well as the relative risk, sensitivity, specificity PPV, NPV, and *P* values for each variable. The *P* values indicate the statistical significance of the results, as determined by two-sided Fisher’s exact test

*CI* confidence interval, *DCI* delayed cerebral ischemia, *hVS* vasospasm with hypoperfusion, n*on-hypo VS* vasospasm without hypoperfusion, *NPV* negative predictive value, *P-CT* cerebral computed tomography with angiography and perfusion, *PPV* positive predictive value, *VS* vasospasm

^a^One patient had missing data for DCI-related infarctions

Based on the criteria of hVS for a positive test result, a subset of 49 patients (89%) who did not show hVS on their screen P-CT scan was identified, and 17 (35%) of these patients eventually developed DCI-related infarction, resulting in an NPV of 0.65 (95% CI 0.50–0.78). Among the six patients who had hVS on their scan, all but one developed DCI-related infarction, resulting in a PPV of 0.83 (95% CI 0.36–1.00, *p* = 0.03) with a risk ratio of 2.4 (95% CI 1.13–5.11, *p* = 0.03) despite induced hypertension, maximal nimodipine, and three patients receiving ERTs. The sensitivity for impending DCI was low at 23% (95% CI 8–45, *p* = 0.03), whereas the specificity was high at 95% (95% CI 36–100, *p* = 0.03). Stratifying by individual days four and eight did not yield statistically significant results.

Using only vasospasm without hypoperfusion as criteria for an inauspicious test result was not significantly associated with DCI-related infarction, but the results showed a higher risk trend than the results of scans without deviating parameters. The high specificity implies that many patients who develop DCI-related infarction also have vasospasm on days four and/or eight.

In a hypothetical situation in which a perfusion scan is not conducted and only CTA is performed, any vasospasm can be considered a positive test result regardless of hypoperfusion. Among 42 patients who tested negative for vasospasm and hypoperfusion on days four and eight, 13 (31%) still developed DCI-related infarction. This resulted in an NPV of 0.69 (95% CI 53–82, *p* = 0.02). Among 14 patients who had vasospasm, with or without hypoperfusion, on screen, nine developed DCI-related infarction and one had missing data, resulting in a PPV of 0.69 (95% CI 39–91, *p* = 0.02) with a risk ratio of 2.2 (95% CI 1.17–4.27, *p* = 0.02) despite induced hypertension, maximal nimodipine, and three patients receiving ERTs. The sensitivity for impending DCI was 41% (95% CI 21–64, *p* = 0.02), whereas the specificity was 88% (95% CI 72–97, *p* = 0.02). Stratifying by individual days four and eight did not yield statistically significant results.

## Discussion

### Summary

In this retrospective study of 56 comatose patients with aSAH receiving P-CT examinations as a screening measure on days four and eight, we demonstrated no improvement from ERTs as a direct result. We also demonstrated that although a positive P-CT scan is likely a strong predictor of impending DCI, a negative P-CT scan provides little to no assurance that the patient will not eventually develop DCI-related infarction.

Objective 1 was to explore the risk-stratifying capabilities. We demonstrated that the NPV of our P-CT protocol (i.e., the proportion of patients with negative screen results who did not develop DCI-related infarction) was 65% for hVS and 69% for vasospasm regardless of hypoperfusion. Therefore, the negative post-test probability for developing DCI-related infarction despite unremarkable scans was 35% and 31%, respectively, which is comparable to the disease’s incidence. We also demonstrated that P-CT often results in late detection of DCI at an irreversible stage, resulting in a sensitivity of 23% for hVS and 41% for vasospasm regardless of hypoperfusion. In total, 40% of the cohort developed DCI-related infarction.

Objective 2 was to explore the clinical implications of P-CT screening in our cohort. Our event-rate analysis found that P-CT screening revealed hVS and allowed interventional treatment in a small group of patients that would have been otherwise undetected; however, all ERT-intervened patients still developed DCI-related infarction.

### Comparison with Literature

Multiple studies have evaluated the implementation of screening protocols including P-CT. In the prospective study by Westermaier et al. [29], a high NPV of 0.99 was for DCI within 72 h in awake patients with aSAH with P-CT screening every three days. In contrast, our comatose study group showed a lower NPV and higher incidence of DCI-related infarction, potentially due to impeded eligibility for neurological examination and worse prognosis in our comatose population, as described in the introduction. The higher incidence of DCI-related infarctions observed in our cohort may partially be due to the differing criteria employed. In our study, we identified DCI-related infarctions within a 6-week period post ictus, whereas Westermaier et al. categorized DCI as new infarctions identified via CT scan within 72 h of a positive neurological examination, TCD, or P-CT result [29]. However, solely considering infarctions from patients with a positive screen result effectively overlooks patients who develop asymptomatic or silent DCI-related infarctions. This definition may be suitable in the clinical context of the study by Westermaier et al., which focused on conscious patients subjected to repeated neurological assessments who were thereby less prone to silent DCI. However, its application to our analysis involving comatose patients would result in an overstated effectiveness of P-CT screening not applicable in a clinical setting of comatose patients because of the exclusion of a significant portion of patients who presented with DCI-related infarctions but exhibited no conspicuous signs in screening silent DCI. Further, it is plausible that alterations detected by P-CT may influence the progression of DCI beyond the immediate 72 h after these changes are observed.

In their retrospective study, Malinova et al. [32] used a P-CT protocol on days 4–5 if a patient was comatose, along with additional scans on clinical indications, and interventions similar to ours, showing improved outcomes, reduced large or multiple infarctions, but no decrease in DCI incidence. This aligns with our findings; however, our study could not confirm improved outcomes or altered DCI severity because of the limited population size and absence of a control group. Furthermore, the study included comatose and awake patients, identifying a significant correlation between higher WFNS scores and severe DCI without stratifying patients based on P-CT guided by neurological examination eligibility vs. P-CT screening only. Our findings suggest that this correlation may be primarily attributed to the lower sensitivity of P-CT screening in the unconscious population [32]. Abdulazim et al. [33] conducted a retrospective study comparing the outcomes of patients with SAH before and after implementing an aggressive monitoring and intervention protocol, including frequent neurological assessments and repeated P-CT scans on days 1, 3–4, and 7–8. They found a significant reduction in DCI and better functional outcomes. However, their protocol differed from ours in that their interventions were more aggressive and evaluated all patients, including those with good grades. As a result, it is challenging to separate the effects of P-CT screening from those of more aggressive interventions and frequent neurological assessments, making it challenging to compare their findings of reduced DCI incidence to the findings in our comatose patient cohort [33].

To our knowledge, a retrospective study by Ditz et al. [20] is the only study evaluating the effects of perfusion screening exclusively in comatose patients. They reported a higher sensitivity for detecting impending DCI than our analysis. However, their study included a baseline P-CT scan, excluded angiography, and had a narrow time frame for DCI-related infarction diagnosis, which makes direct comparison difficult [20]. The lack of a standardized hypoperfusion definition further complicates comparisons and is a limitation of retrospective studies. Although hypoperfusion association with DCI is well established [34], CTA can easily be performed sequentially and help link impending DCI with a known pathology for which current interventions exists [22]. Furthermore, our study found only one case of hypoperfusion without vasospasm, and our NPV analysis demonstrated that the absence of vasospasm, regardless of hypoperfusion, had better prognostic certainty than the absence of hVS and hypoperfusion alone (Table 3).

The incidence of DCI-related infarction in our study was higher than the literature otherwise suggests for patients with aSAH [2, 5]. However, the cohort was largely patients with poor-grade aSAH with high WFNS, Hunt and Hess, and Fisher scores. A 2022 meta-analysis found the event rate of DCI in studies including poor-grade patients to be 0.40 (95% CI 0.24–0.56), in line with our results [5].

Determining when to perform routine P-CT scans in comatose patients is difficult because of the complex and varied onset of DCI and the limited window for treatment. The literature suggests that the highest risk of hVS is on days 4–14 [2]. This is in line with the results of our study, although conclusions on which days carry the highest risk cannot be made from this study, as those results are biased by the large number of scans done on days four and eight. In our study, many patients suffered infarction between days four and eight, but an even larger group suffered infarction between day eight and the 6-week mark. It is possible that the underlying cause of the infarction in the latter group was initiated on days 8–14. It could also be due to an ongoing neuroinflammatory reaction or other possible mechanisms described in the literature [12].

### Limitations

This study has some limitations that should be considered when interpreting the results. Firstly, no functional outcome or quality of life was included, which is critical to evaluating a screening modality. Additionally, the sample size was small, and therefore the estimate of the relative risks of mortality and DCI may not be precise. Although the results were statistically significant, extrapolating these findings to clinical practice may be uncertain because of the wide CIs and the possibility of interventions affecting outcomes.

The retrospective design of our study, along with certain limitations in our scanning protocol, could have potentially led to an overestimation or underestimation of DCI-related infarction. Notably, the absence of a baseline scan before the initial P-CT screening and the lack of scans at the 6-week mark posed significant challenges. Specifically, it was difficult to distinguish DCI-related infarction from other causes on the day four P-CT scans. Moreover, the absence of routine 6-week scans might have resulted in undetected late-developing DCI-related infarction. It should also be noted that our study predominantly used CT scans, which have a lower sensitivity for DCI-related infarct detection compared to magnetic resonance imaging (MRI) [35]. Despite these limitations, we undertook a meticulous process for the identification and differentiation of DCI-related and non-DCI-related infarcts. This process involved review of patient charts and the use of CTC, perfusion, and CTA scans, which were initially assessed and described by neuroradiologists and later retrospectively analyzed by a vascular neurosurgeon at the time of data extraction, excluding infarction not attributable to DCI.

The clinical protocol implemented in our study, which involves the prompt replacement of propofol with midazolam during prolonged sedation, impeded the ability for wake-up calls in numerous patients. This limited our ability to detect signs of clinical deterioration, which could indicate impending DCI and ultimately could have resulted in a greater number of additional P-CT scans and subsequent interventions.

Additionally, the study design is limited by the lack of a control group, so the outcome comparison between groups is likely biased by interventions resulting from vasospasm and hVS. This may have resulted in a bias toward higher sensitivity, lower specificity, and lower PPV, as some cases of DCI-related infarction may have been prevented by interventions. Therefore, NPV may be a more reliable outcome measure based on patients not subjected to interventions.

Lastly, our statistical analysis is based on the assumption that hVS is a perfect surrogate for impending DCI; however, infarct not attributable to P-CT-detected hVS could be responsible for DCI-related infarction. As previously mentioned, DCI is likely a complex and multifactorial disease; hence, our study method may overestimate the incidence of DCI-related infarction due to hVS. On the other hand, the pathophysiology behind DCI-related infarction is heterogenous and poorly understood, and it is possible that perturbations seen on P-CT will impact the development of DCI-related infarction further in the future than just 48 or 72 h after the P-CT changes appear, which some previously mentioned studied applied for their diagnostic cutoff.

### Perspectives

Increasing the frequency of scans to increase the detection rate presents trade-offs between benefits and potential risks. An easy way to increase sensitivity and NPV would be to increase the number of P-CT scans. However, additional scans raises concerns regarding radiation burden and renal impairment, which limit the number of occasions on which P-CT can be performed to screen for vasospasm and impending DCI [36]. Radiation and contrast nephropathy can be circumvented using MRI methods instead of CT. However, this modality has some additional disadvantages. MRI is more expensive and less readily available than CT, especially in emergency settings where time is critical. Additionally, MRI may not be suitable for all patients, including those with metallic implants. Thus, the decision to use MRI as an alternative to P-CT should be carefully considered based on the specific needs of each patient and the resources available. An argument can be made that the risk/benefit ratio is more favorable in poor-grade patients with a bad prognosis when the implications of not diagnosing DCI early can be severe. In addition to scanning on days four and eight, a third P-CT on day six could be implemented, with the hope to improve the rate of early DCI detection in the group diagnosed on day eight but who did not show signs of injury on day four. Also, the literature suggests that vasospasm is maximal on days six to eight [37]. MRI studies suggest DCI-related infarction after SAH is usually bilateral and multifocal, often involving the frontal lobes [38]. This constitutes a challenge for the P-CT modality, as perfusion deficits bilaterally can be masked, giving a rationale for obtaining a baseline P-CT. Further, a study by Rodriguez-Régent et al. found variations of mean transit time and cerebral blood flow values between day zero and day four to predict DCI [39]. For the large group of patients developing DCI-related infarction post day eight, it is difficult to access when the infarction occurs. Therefore, it is challenging to extrapolate which days post day eight the patient is most at risk. Results from our additional P-CT scans suggest that patients with clinical deterioration, as found on multimodal monitoring, showed hVS in 5 of 15 scans, occurring between days 9 and 15.

The combination of angiography and perfusion scans (P-CT) appears to be more effective than using either method alone in identifying high-risk patients. Furthermore, angiography allows visualization of posterior circulation whereas perfusion maps may be inconclusive, and although not statistically significant, our results of vasospasm without hypoperfusion skew toward a higher risk of developing DCI-related infarction regardless of the day. Based on this, we cannot rule out that vasospasm might be an independent risk factor, even in the absence of hypoperfusion, pointing to the complex multifactorial pathology of DCI. However, angiography should not stand alone, as not all vasospasms are reliable indicators of impending DCI, and the risks of ERTs likely outweigh the benefits for asymptomatic vasospasm. Our results suggest that vasospasm in the absence of hypoperfusion has the weakest correlation with DCI-related infarction and mortality of the three possible scan results (Table 3), which aligns with the existing literature described in the Introduction section. Our study was not designed to investigate whether treating all vasospasms with induced hypertension and maximal nimodipine improves outcomes, and it failed to show improved outcomes of ERTs for hVS detected on screen. Nonetheless, based on our results, it can be hypothesized that hVS can guide further interventions for those patients who will likely benefit most from it.

Our results suggest that P-CT on days four and eight after aSAH onset is unsuitable for screening because of low sensitivity and low NPV. As described previously, it seems inappropriate to increase NPV and sensitivity just by increasing the number of P-CT scans performed. Hence, the high deterioration rate highlights the need for continuous monitoring.

It may be possible to increase NPV and sensitivity by using multimodal monitoring methods to guide additional P-CT scans. For instance, monitoring modalities such as S100B, TCD, ICP, continuous electroencephalography (EEG), brain tissue oxygen monitoring, and cerebral microdialysis (CMD) can indicate signs of deterioration. In such cases, P-CT can confirm the suspicion before advancing to the more invasive DSA procedure.

CMD allows continuous bedside monitoring of chemical substances related to energy metabolism and has recently been assessed as a predictor and diagnostic tool for DCI in comatose patients. According to a systematic review, brain tissue oxygen partial pressure, lactate pyruvate ratio, and glutamate concentration can diagnose impending DCI [40]. However, the drawback of these methods is that they are highly regional and limited to the “area at risk,” which does not cover the multifocal nature of DCI. To the authors’ knowledge, no studies have prospectively analyzed impact of CMD-guided interventions [41].

Another continuous monitoring modality that is believed to be highly sensitive to cerebral ischemia is EEG [42]. Although continuous EEG monitoring is technically cumbersome and labor-intensive, it is a global monitoring method. A prospective study including patients with poor-grade aSAH suggests that the number of patients needed to monitor to predict one additional case of DCI prior to clinical symptoms is between three and seven [43], and with the rapid development of artificial intelligence, this could be improved. However, further studies, including event-rate analysis, are necessary to document the impact of EEG on patient outcomes and the effect of potential specific interventions beyond the treatment of hVS.

## Conclusions

A positive hVS result is likely a strong predictor of future DCI-related infarction despite interventions, but the absence of hVS in P-CT examinations on days four and eight was not a reliable indicator that the patient would not later develop DCI-related infarction.

In line with existing literature, our result shows that a positive P-CT result accurately predicts impending DCI-related infarction with a high relative risk, specificity, and PPV. This implies that P-CT could be used as a confirmatory test prior to more invasive interventions. However, P-CT screening on days four and eight in unconscious patients with aSAH failed to timely detect impending DCI in deteriorating patients, as evidenced by low sensitivity, low NPV, and a negative post-test probability practically equal to the incidence of DCI. Furthermore, all three patients who received ERTs as a direct result of screening developed DCI-related infarction. Whether increased detection of vasospasm and/or hypoperfusion and resulting interventions improve outcomes remains unknown, and prospective studies with functional outcome measures are warranted for comatose patients.

Based on our analysis, we cannot recommend P-CT as a screening modality on days four and eight alone for screening purposes in this group of patients. P-CT is likely best indicated by continuous multimodal monitoring and used as a confirmatory test prior to more invasive interventions.

## References

[1] Muehlschlegel S. Subarachnoid Hemorrhage. Continuum (Minneap Minn) 2018;24(6):1623–1657. (In Eng). 10.1212/con.0000000000000679. 10.1212/CON.0000000000000679 30516599

[2] Neifert SN, Chapman EK, Martini ML (2021). Aneurysmal subarachnoid hemorrhage: the last decade. Transl Stroke Res.

[3] Etminan N, Chang H-S, Hackenberg K (2019). Worldwide incidence of aneurysmal subarachnoid hemorrhage according to region, time period, blood pressure, and smoking prevalence in the population: a systematic review and meta-analysis. JAMA Neurol.

[4] Yamaki VN, Cavalcanti DD, Figueiredo EG (2019). Delayed ischemic neurologic deficit after aneurysmal subarachnoid hemorrhage. Asian J Neurosurg.

[5] Rigante L, van Lieshout JH, Vergouwen MDI, et al. Time trends in the risk of delayed cerebral ischemia after subarachnoid hemorrhage: a meta-analysis of randomized controlled trials. Neurosurg Focus 2022;52(3):E2. (In Eng). 10.3171/2021.12.Focus21473. 10.3171/2021.12.FOCUS21473 35231892

[6] Al-Mufti F, Amuluru K, Changa A (2017). Traumatic brain injury and intracranial hemorrhage-induced cerebral vasospasm: a systematic review. Neurosurg Focus.

[7] Dorsch N (2011). A clinical review of cerebral vasospasm and delayed ischaemia following aneurysm rupture. Acta Neurochir Suppl.

[8] Macdonald RL (2014). Delayed neurological deterioration after subarachnoid haemorrhage. Nat Rev Neurol.

[9] Jacobsen A, Nielsen TH, Nilsson O, Schalen W, Nordstrom CH (2014). Bedside diagnosis of mitochondrial dysfunction in aneurysmal subarachnoid hemorrhage. Acta Neurol Scand.

[10] Chen S, Wu H, Tang J, Zhang J, Zhang JH. Neurovascular events after subarachnoid hemorrhage: focusing on subcellular organelles. Acta Neurochir Suppl 2015;120:39–46. (In Eng). 10.1007/978-3-319-04981-6_7. 10.1007/978-3-319-04981-6_7 PMC4373344 25366597

[11] Weiland J, Beez A, Westermaier T, Kunze E, Siren AL, Lilla N. Neuroprotective strategies in aneurysmal subarachnoid hemorrhage (aSAH). Int J Mol Sci 2021;22(11). 10.3390/ijms22115442. 10.3390/ijms22115442 PMC8196706 34064048

[12] Geraghty JR, Testai FD (2017). Delayed cerebral ischemia after subarachnoid hemorrhage: beyond vasospasm and towards a multifactorial pathophysiology. Curr Atheroscler Rep.

[13] Tulamo R, Frösen J, Hernesniemi J, Niemelä M. Inflammatory changes in the aneurysm wall: a review. J Neurointerv Surg 2010;2(2):120–30. (In Eng). 10.1136/jnis.2009.002055. 10.1136/jnis.2009.002055 21990591

[14] Dankbaar JW, Rijsdijk M, van der Schaaf IC, Velthuis BK, Wermer MJH, Rinkel GJE. Relationship between vasospasm, cerebral perfusion, and delayed cerebral ischemia after aneurysmal subarachnoid hemorrhage. Neuroradiology 2009;51(12):813–819. (In English). 10.1007/s00234-009-0575-y. 10.1007/s00234-009-0575-y PMC2773037 19623472

[15] Francoeur CL, Mayer SA (2016). Management of delayed cerebral ischemia after subarachnoid hemorrhage. Crit Care.

[16] Vergouwen MD, Vermeulen M, van Gijn J, et al. Definition of delayed cerebral ischemia after aneurysmal subarachnoid hemorrhage as an outcome event in clinical trials and observational studies: proposal of a multidisciplinary research group. Stroke 2010;41(10):2391–5. (In Eng). 10.1161/strokeaha.110.589275. 10.1161/STROKEAHA.110.589275 20798370

[17] Hoh BL, Ko NU, Amin-Hanjani S (2023). 2023 Guideline for the management of patients with aneurysmal subarachnoid hemorrhage: a guideline from the American Heart Association/American Stroke Association. Stroke.

[18] Chamorro C, de Latorre FJ, Montero A (1996). Comparative study of propofol versus midazolam in the sedation of critically ill patients: results of a prospective, randomized, multicenter trial. Crit Care Med.

[19] Ivanidze J, Sanelli PC. Vasospasm: role of Imaging in detection and monitoring treatment. Neuroimaging Clin N Am 2021;31(2):147–155. (In Eng). 10.1016/j.nic.2021.01.004. 10.1016/j.nic.2021.01.004 33902870

[20] Ditz C, Hartlieb M, Neumann A (2021). Routine use of perfusion computed tomography for the detection of delayed cerebral ischemia in unconscious patients after aneurysmal subarachnoid hemorrhage. Acta Neurochir.

[21] Mir DI, Gupta A, Dunning A, et al. CT perfusion for detection of delayed cerebral ischemia in aneurysmal subarachnoid hemorrhage: a systematic review and meta-analysis. AJNR Am J Neuroradiol. 2014;35(5):866–71. (In Eng). 10.3174/ajnr.A3787. 10.3174/ajnr.A3787 PMC4159608 24309123

[22] Dankbaar JW, Rijsdijk M, van der Schaaf IC, Velthuis BK, Wermer MJ, Rinkel GJ. Relationship between vasospasm, cerebral perfusion, and delayed cerebral ischemia after aneurysmal subarachnoid hemorrhage. Neuroradiology 2009;51(12):813–9. (In Eng). 10.1007/s00234-009-0575-y. 10.1007/s00234-009-0575-y PMC2773037 19623472

[23] Sanelli PC, Ugorec I, Johnson CE, et al. Using quantitative CT perfusion for evaluation of delayed cerebral ischemia following aneurysmal subarachnoid hemorrhage. AJNR Am J Neuroradiol 2011;32(11):2047–53. (In Eng). 10.3174/ajnr.A2693. 10.3174/ajnr.A2693 PMC3237788 21960495

[24] Aralasmak A, Akyuz M, Ozkaynak C, Sindel T, Tuncer R (2009). CT angiography and perfusion imaging in patients with subarachnoid hemorrhage: correlation of vasospasm to perfusion abnormality. Neuroradiology.

[25] Shi D, Jin D, Cai W (2020). Serial low-dose quantitative CT perfusion for the evaluation of delayed cerebral ischaemia following aneurysmal subarachnoid haemorrhage. Clin Radiol.

[26] Killeen RP, Mushlin AI, Johnson CE, et al. Comparison of CT perfusion and digital subtraction angiography in the evaluation of delayed cerebral ischemia. Acad Radiol 2011;18(9):1094–100. (In Eng). 10.1016/j.acra.2011.04.004. 10.1016/j.acra.2011.04.004 PMC3152644 21652232

[27] Lui YW, Tang ER, Allmendinger AM, Spektor V. Evaluation of CT perfusion in the setting of cerebral ischemia: patterns and pitfalls. AJNR Am J Neuroradiol 2010;31(9):1552–63. (In Eng). 10.3174/ajnr.A2026. 10.3174/ajnr.A2026 PMC7965002 20190208

[28] Wintermark M, Ko NU, Smith WS, Liu S, Higashida RT, Dillon WP. Vasospasm after subarachnoid hemorrhage: utility of perfusion CT and CT angiography on diagnosis and management. AJNR Am J Neuroradiol 2006;27(1):26–34. (In Eng). PMC7976085 16418351

[29] Westermaier T, Pham M, Stetter C, et al. Value of transcranial Doppler, perfusion-CT and neurological evaluation to forecast secondary ischemia after aneurysmal SAH. Neurocrit Care 2014;20(3):406–12. (In Eng). 10.1007/s12028-013-9896-0. 10.1007/s12028-013-9896-0 23982597

[30] Dansk-Neurokirurgisk-Selskab. Vejledning: aneurysmal subarachnoidalblødning - Diagnose og Behandling. 1 feb 2022.

[31] Balança B, Bouchier B, Ritzenthaler T. The management of delayed cerebral ischemia after aneurysmal subarachnoid hemorrhage. Rev Neurologique 2022;178(1–2):64–73. (In English). 10.1016/j.neurol.2021.11.006. 10.1016/j.neurol.2021.11.006 34961603

[32] Malinova V, Döring K, Psychogios MN, Rohde V, Mielke D. Impact of implementing an elaborated CT perfusion protocol for aneurysmal SAH on Functional outcome: CTP protocol for SAH. AJNR Am J Neuroradiol 2021;42(11):1956–61. (In eng). 10.3174/ajnr.A7279. 10.3174/ajnr.A7279 PMC8583263 34556476

[33] Abdulazim A, Küppers C, Hackenberg KAM (2022). Multidisciplinary and standardized management of patients with delayed cerebral ischemia after aneurysmal subarachnoid hemorrhage. Acta Neurochir.

[34] Cremers CHP, van der Schaaf IC, Wensink E (2014). CT perfusion and delayed cerebral ischemia in aneurysmal subarachnoid hemorrhage: a systematic review and meta-analysis. J Cereb Blood Flow Metab.

[35] Korbakis G, Prabhakaran S, John S (2016). MRI detection of cerebral infarction in subarachnoid hemorrhage. Neurocrit Care.

[36] Imanishi Y, Fukui A, Niimi H, et al. Radiation-induced temporary hair loss as a radiation damage only occurring in patients who had the combination of MDCT and DSA. Eur Radiol 2005;15(1):41–6. (In Eng). 10.1007/s00330-004-2459-1. 10.1007/s00330-004-2459-1 15351903

[37] Weir B, Grace M, Hansen J, Rothberg C. Time course of vasospasm in man. J Neurosurg. 1978;48(2):173–78. (In English). 10.3171/jns.1978.48.2.0173. 10.3171/jns.1978.48.2.0173 624965

[38] Rabinstein AA, Friedman JA, Weigand SD, et al. Predictors of cerebral infarction in aneurysmal subarachnoid hemorrhage. Stroke (1970) 2004;35(8):1862–66. (In English). 10.1161/01.STR.0000133132.76983.8e. 10.1161/01.STR.0000133132.76983.8e 15218156

[39] Rodriguez-Régent C, Hafsa M, Turc G, et al. Early quantitative CT perfusion parameters variation for prediction of delayed cerebral ischemia following aneurysmal subarachnoid hemorrhage. Eur Radiol 2016;26(9):2956–63. (In Eng). 10.1007/s00330-015-4135-z. 10.1007/s00330-015-4135-z 26670321

[40] Veldeman M, Albanna W, Weiss M, et al. Invasive multimodal neuromonitoring in aneurysmal subarachnoid hemorrhage: a systematic review. stroke 2021:Strokeaha121034633. (In Eng). 10.1161/strokeaha.121.034633. 10.1161/STROKEAHA.121.034633 34304602

[41] Skjøth-Rasmussen J, Schulz M, Kristensen SR, Bjerre P. Delayed neurological deficits detected by an ischemic pattern in the extracellular cerebral metabolites in patients with aneurysmal subarachnoid hemorrhage. J Neurosurg 2004;100(1):8–15. (In Eng). 10.3171/jns.2004.100.1.0008. 10.3171/jns.2004.100.1.0008 14743906

[42] Yu Z, Wen D, Zheng J (2019). Predictive accuracy of alpha-delta ratio on quantitative electroencephalography for delayed cerebral ischemia in patients with aneurysmal subarachnoid hemorrhage: meta-analysis. World Neurosurg.

[43] Rosenthal ES, Biswal S, Zafar SF (2018). Continuous electroencephalography predicts delayed cerebral ischemia after subarachnoid hemorrhage: a prospective study of diagnostic accuracy. Ann Neurol.

